# Estimating Seasonal Nitrogen Removal and Biomass Yield by Annuals with the Extended Logistic Model

**DOI:** 10.1371/journal.pone.0095934

**Published:** 2014-04-22

**Authors:** Richard V. Scholtz, Allen R. Overman

**Affiliations:** Agricultural & Biological Engineering Department, University of Florida, Gainesville, Florida, United States of America; University of Vigo, Spain

## Abstract

The Extended Logistic Model (ELM) has been previously shown to adequately describe seasonal biomass production and N removal with respect to applied N for several types of annuals and perennials. In this analysis, data from a corn (*Zea mays* L.) study with variable applied N were analyzed to test hypotheses that certain parameters in the ELM are invariant with respect to site specific attributes, like environmental conditions and soil type. Invariance to environmental conditions suggests such parameters may be functions of the crop characteristics and certain other management practices alone (like plant population, planting date, harvest date). The first parameter analyzed was Δ*b*, the difference between the N uptake shifting parameter and the biomass shifting parameter. The second parameter tested was *N_cm_*, the maximum N concentration. Both parameters were shown to be statistically invariant, despite soil and site differences. This was determined using analysis of variance with normalized nonlinear regression of the ELM on the data from the study. This analysis lends further evidence that there are common parameters involved in the ELM that do not rely on site-specific or situation-specific factors. More insight into the derivation of, definition of, and logic behind the various parameters involved in the model are also given in this paper.

## Introduction

Effective water and nutrient management plays an essential role in future attempts at sustainable agricultural production. As the world’s population continues to grow, the potable water supply is limited and must be guarded from unnecessary withdrawals and contamination from excessive nutrient loads. Strict monitoring and exercises in groundwater modeling of all agricultural operations is cost prohibitive. It therefore becomes necessary to investigate crop nutrient removal from their environment, and to adopt management procedures and rules that are based on a sound scientific foundation.

Overman et al. first proposed the logistic model as a nutrient management tool to describe seasonal biomass yield dependence of forage grasses on applied N [1]. The original application of the logistic model to plant biomass production was based on inductive reasoning, a process where inferences are made from “real world” observations [2]. While inductive reasoning is innately a more empirical method of model development, all models, no matter the complexity, have some element of empiricism [3]. It is because of the application to the “real world,” that engineering and the applied sciences are, for practical reasons, inherently more empirical. The logistic model was extended to include seasonal plant N uptake (removal from the environment) dependence on applied N by forage grasses [4] and then for annuals, like corn [5]. The ELM is a five parameter, non-linear, parametric model that is capable of describing the seasonal biomass yields, N uptake, and N concentration with respect to applied N. Work conducted over the years has indicated that the ELM can effectively describe both annual [6]–[11] and perennial [8], [12]–[20] crops, for a wide range of nutrient inputs.

The ELM, begins with the simple logistic expression that relates N uptake, *N_u_*, to the N applied, *N*, which is given by the following relationship:
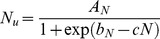
(1)where *A_N_* is the relative maximum N uptake in kg ha^−1^, *b_N_* is the dimensionless N uptake shifting parameter, and *c* is the applied N response parameter given in ha kg^−1^. The phase relationship between biomass production, *Y*, and N uptake, *N_u_*, is given by
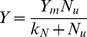
(2)where *Ym* is the maximum potential biomass production in Mg ha^−1^, and *k_N_* is the N uptake response parameter in kg ha^−1^. The transformation of Eq. (1) by using Eq. (2) yields the following logistic expression:
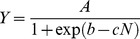
(3)that relates biomass production to applied N, where *A* is the relative maximum biomass production in Mg ha^−1^, *b* is the biomass yield shifting parameter, and *c* is the same applied N response parameter that applies to the N uptake logistic equation. The parameters *A_N_*, *A*, *b_N_*, *b*, and *c* are currently key parameters used with the ELM, and are the easiest to determine from regression analysis.

From the transformation of Eq. (1) into Eq. (3), the relative maximum biomass yield parameter can be written in terms of the maximum potential biomass yield parameter, the relative maximum N uptake parameter, and the N uptake response parameter.
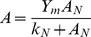
(4)


The biomass shifting parameter can be written in terms of the N uptake shifting parameter, the relative maximum N uptake parameter, and the N uptake response coefficient.
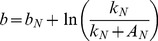
(5)


The difference between the shifting parameter for N uptake and the biomass yield can be written as the following:
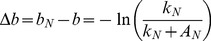
(6)


N concentration is simply defined as the ratio of N uptake to biomass production. This leads to the following relationship:
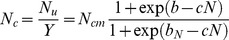
(7)


The N concentration model suggests that as N applied is increased to exorbitant levels, there is a maximum limit to the N concentration, *N_cm_*. This maximum limit is simply the ratio of the relative maximum N uptake with respect to applied N, *A_N_*, to the relative maximum biomass production with respect to applied N, *A*.
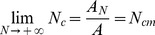
(8)


It seems logical to suggest that as the background amount of N present in the soil is decreased, the N concentration would be reduced to some prescribed lower limit, as there would be lower limit on the percent of proteins present in a given crop to sustain any growth. Mathematically, the model suggests that this lower limit of N concentration, *N_cl_*, is a function of the maximum concentration and the difference between the N uptake shifting parameter and the biomass shifting parameter.
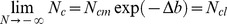
(9)


Also, this lower limit of N concentration, *N_cl_*, can be found by taking the ratio of the N uptake response coefficient to the maximum potential biomass production parameter.
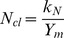
(10)


From the phase relationship between biomass production and N uptake, Eq. (2), N concentration can be found from the following equation with respect to N uptake:
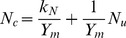
(11)


This predicts a linear relationship between N concentration, *N_c_*, and N uptake, *N_u_*. The line should have a slope equal to the inverse of the maximum potential biomass production and an intercept that equals the ratio of the N uptake response parameter to maximum potential biomass production. As this is a phase relationship, this is a functional segment that is bounded between N uptake values from 0 to the peak of *A_N_*, and between N concentration values between *N_cl_* and *N_cm_*.

From the earlier work of Overman et al. [5], it has been shown that for a given site the applied N response parameter, *c*, and the N uptake intercept parameter, *b_N_*, and the biomass intercept parameter, *b*, are not unique to the ELM when applied to grain or the whole plant. Meaning that the harvest index is constant for a given site. Their analysis showed that the only differences between the grain and the whole plant appear in the relative maximum N uptake parameter, *A_N_*, and the relative maximum biomass production parameter, *A* [5]. Because the *b_N_*, *b*, and *c* parameters were shown to be constant for both grain fraction and total biomass production, all the differences in both grain N uptake and biomass production, and the differences in the total plant N uptake and biomass production can be estimated with seven model parameters and a value for the seasonal amount of N applied. This is a comparative reduction of three parameters when *b_N_*, *b*, and *c* are not held constant between grain and total plant biomass production.

The goal of this work is to continue to elevate the Extended Logistic Model (ELM) beyond the empiricism of it nascent beginnings and achieve a balance between what can be measured and what should be modeled, as called for by Montieth [21]. The intent is to shed new light on the significance of parameters used in the ELM and to contribute to the search for commonality among parameters. Normalized non-linear regression and analysis of variance (ANOVA) were used to show the invariance of two model parameters with respect to environmental differences, namely soil type and water availability.

## Methods

### Data Set

This analysis uses data collected by Eugene Kamprath from a corn (Pioneer 3320) N-rate field study that was conducted at three regional research stations in North Carolina from 1981 to 1984. A detailed explanation of the field experiment has been previously reported [22]. Supplemental irrigation was provided at the Clayton experiment station for the well-drained Dothan loamy fine sand (*fine-loamy, siliceous, thermic Plinthic Kandiudults*), at a rate of 10 to 12 cm a season, except for 1982 when no additional water was supplied. No irrigation was provided at the Kinston station for the well-drained Goldsboro sandy loam (*fine-loamy, siliceous, thermic Aquic Paleudults*). At the Plymouth experiment station, no irrigation was provided for the poorly-drained Portsmouth very fine sandy loam (*fine-loamy over sandy or sandy-skeletal mixed, thermic Typic Umbraquults*). The experiments at each station were set up as a RCB design, with four replications. Both total plant and grain fraction biomass were sampled, and every year the experiment was conducted at a new location within the same soil type at each station. This was to limit the impact on the experiment of any residual N in the soil from the previous year. The fertilizer treatments were applied in the form of NH_4_NO_3_ at rates of 0, 56, 112, 168, and 224 kg ha^−1^ of N. Average values over the four year period were combined for each of the different treatments and the model parameters were evaluated based on those combined averages.

### Normalization

Parameters *A_N_* and *A* for grain, *A_N_* and *A* for total plant, and *b_N_*, *b* and *c* for each site are determined simultaneously, using Newton-Raphson non-linear regression. A detailed description of Newton-Raphson non-linear regression of logistic equation can be found in Overman and Scholtz [8]. The attempt of this methodology is to consistently distribute the standard error amongst all those parameters for further analysis. Because of the unit and an order of magnitude difference between biomass and N uptake parameter values, as well as a subsequent order of magnitude difference between grain and total plant parameter values, a normalization routine is also employed. The error sum of squares for each individual site is initially written as
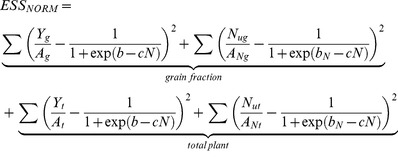
(12)where the total normalized error is resultant from the sum of the normalized error from the three sites. For this study the initial Hessian matrix is 21 by 21 elements and paired with a 21 element Jacobian vector. As a result of the normalization procedure, performing the Newton-Raphson procedure can diverge more readily than a non-normalized procedure. Because of this, it is important to establish a reasonable initial guess for each parameter. First, individual logistic response are evaluated for each data set from all sites, for grain biomass, grain N uptake, for total plant biomass, and total plant N uptake. This results in six *A* values, six *A_N_* values, six *b* values, six *b_N_* values, and 12 values for the *c* parameter. Using the fact that it can be shown for two straight lines
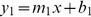
(13)and
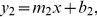
(14)who share the same sampling of the independent variable, the best fit single slope shared between them is given by
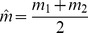
(15)and the corresponding intercepts become
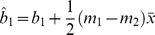
(16)and
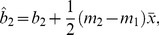
(17)the initial guess for the *c* parameter is the average of all 12 values, and the initial guess for each value of bN can be found from
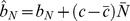
(18)and for each value of *b* can be found from



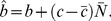
(19)The problem is bounded between the maximum and minimum values of the *c* parameter and each value of *b_N_* is bounded between
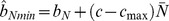
(20)and
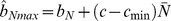
(21)and each value of *b* is bounded between
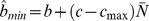
(22)and
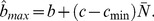
(23)


### Analysis

The first hypothesis of this analysis is that the difference between N uptake intercept parameter and the biomass intercept parameter, *Δb*, is invariant with respect to the differences in soil type and water availability for a given variety of an annual crop. Note that there is no attempt in this work to identify the effects of water availability or site characteristics on the ELM parameters, but to determine which are invariant to those characteristics. For this analysis, the same genetic line of corn is propagated by seeding and harvested at the same relative age. The second hypothesis is that maximum N concentration, *N_cm_*, is also invariant with respect to the differences in soil type and water availability. A consequence of both hypotheses being affirmed is that the lower limit to the N concentration, *N_cl_*, in the same annual crop is also invariant with respect to soil type and water availability. Parameters were estimated by minimization of the normalized error sum of squares, and analysis of variance (ANOVA) was used to determine the validity of both hypotheses.

For the analysis of variance, three scenarios or modes were used, each with a targeted reduction in the number of parameters used in the ELM to describe the corn data in the Kamprath study. Mode I had 21 separate parameters that were estimated by minimization of the normalized error sum of squares. In Mode I, there are individual values for *A*, and *A_N_*, for both grain and for total plant, and corresponding values for *b*, *b_N_*, and *c* at each of the three sites. For Mode II, the number of parameters estimated dropped to 19, because the *Δb* parameter was held constant across the three sites. For Mode III, the *Δb* and the *N_cm_* parameters were both held constant across the three sites, reducing the number of estimated parameters to 15.

Nonlinear Coefficients of Determination [23] (Nash-Sutcliffe Model Efficiency Coefficient [24]) will be provided for grain and total plant N uptake and for grain and total plant biomass production just as a relative comparison of fit.

## Results

Table 1 contains the summary of the analysis of variance test. The comparison between Modes I and II leads to an increase the *degrees of freedom* to 41 and results in a variance ratio of 0.940. Because the critical F(2,39,95%) value is 3.24, it is concluded that there is no significant difference between the two modes. Thus in this study the Δ*b* parameter is invariant to all soil and site differences.

**Table 1:** Analysis of variance for model parameters for corn grain and total plant biomass production and for corn grain and total plant N uptake, grown on three different soils.

Mode	ParametersEstimated	Degrees ofFreedom	Normalized ResidualSum of Squares	Normalized MeanSum of Squares	*F* Value
I	21	39	0.0247	0.000633	––––
II, Common Δ*b*.	19	41	0.0259	0.000631	––––
II–I	––	2	0.00119	0.000595	0.940
III, Common *Δb, & N_cm_*.	15	45	0.0267	0.000594	––––
III–I	––	6	0.00202	0.000337	0.533

Also from Table 1, the comparison between Modes I and III results in an increase the *degrees of freedom* to 45, and in a variance ratio of 0.533. With a critical F(6,39,95%) value of 2.34, not only is the Δ*b* parameter is invariant, but so are the total plant and grain *N_cm_* parameters. Thus, the soil, the field conditions, the environmental constraints, and even water availability play no role in either is the Δ*b* or the two *N_cm_* parameters. This leads to an invariance in the total plant and grain *N_cl_* parameters, by virtue of Eq. (9).

**Figure 1:**
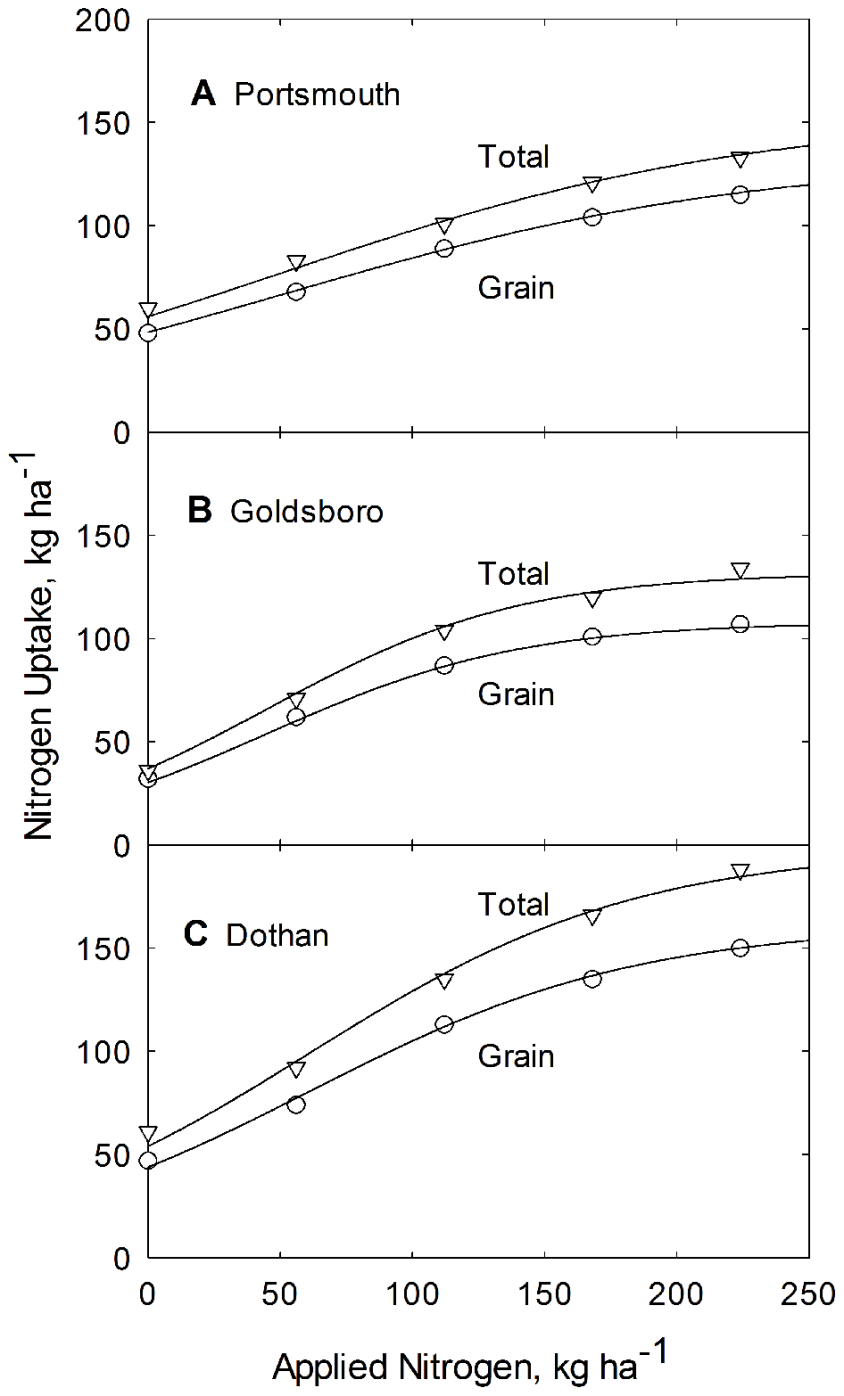
Dependence of grain and total plant N uptake on applied N for corn grown at the Plymouth (A), Kinston (B), and Clayton (C) experiment stations in North Carolina. Curves are constructed from Eq. 1 and from parameters listed in Table 2.

The dependence of grain and whole plant N uptake on applied N at harvest is represented by Figure 1 for the three soil types. In general there is good agreement between the model line and the data. The resulting N uptake model lines (depicted in Figure 1), are generated from Eq. (1), using parameter values for *b_N_* and *c* from Table 2 and values for *A_Ng_* and *A_Nt_* found in Table 3. Equation specific Non-linear Coefficient of Determination values and Error Sum of Squares are provided in Table 4.

**Table 2:** Standard logistic model parameters invariant to corn grain and total plant biomass production and for corn grain and total plant N uptake, grown on three different soils.

Soil	Component	Parameter	Estimate
Dothan	Biomass	*b*	0.319
	N Uptake	*b_N_*	0.978
	Both	*c*, ha kg^−1^	0.0161
Goldsboro	Biomass	*b*	0.282
	N Uptake	*b_N_*	0.941
	Both	*c*, ha kg^−1^	0.0209
Portsmouth	Biomass	*b*	−0.105
	N Uptake	*b_N_*	0.555
	Both	*c*, ha kg^−1^	0.0111

**Table 3:** Standard logistic model parameters specific to corn grain and total plant biomass production and for corn grain and total plant N uptake, grown on three different soils.

Soil	Component	Parameter	Plant Fraction	Estimate
Dothan	Biomass	*A_g_*, Mg ha^−1^	Grain	11.5
		*A_t_*, Mg ha^−1^	Total Plant	21.5
	N Uptake	*A_Ng_*, kg ha^−1^	Grain	161
		*A_Nt_*, kg ha^−1^	Total Plant	198
Goldsboro	Biomass	*A_g_*, Mg ha^−1^	Grain	7.76
		*A_t_*, Mg ha^−1^	Total Plant	14.4
	N Uptake	*A_Ng_*, kg ha^−1^	Grain	108
		*A_Nt_*, kg ha^−1^	Total Plant	132
Portsmouth	Biomass	*A_g_*, Mg ha^−1^	Grain	9.50
		*A_t_*, Mg ha^−1^	Total Plant	16.7
	N Uptake	*A_Ng_*, kg ha^−1^	Grain	133
		*A_Nt_*, kg ha^−1^	Total Plant	154

**Table 4:** Standard statistical measures of fit, based on specific component and plant fraction data.

Soil	Component	Plant Fraction	Coefficient of Determination	Specific Error Sum of Squares
Dothan	Biomass	Grain	0.984	0.523
		Total Plant	0.969	2.361
	N Uptake	Grain	0.997	24.5
		Total Plant	0.993	83.9
Goldsboro	Biomass	Grain	0.992	0.145
		Total Plant	0.983	0.468
	N Uptake	Grain	0.998	7.81
		Total Plant	0.993	35.2
Portsmouth	Biomass	Grain	0.977	0.169
		Total Plant	0.985	0.592
	N Uptake	Grain	0.999	5.01
		Total Plant	0.991	19.4

**Figure 2:**
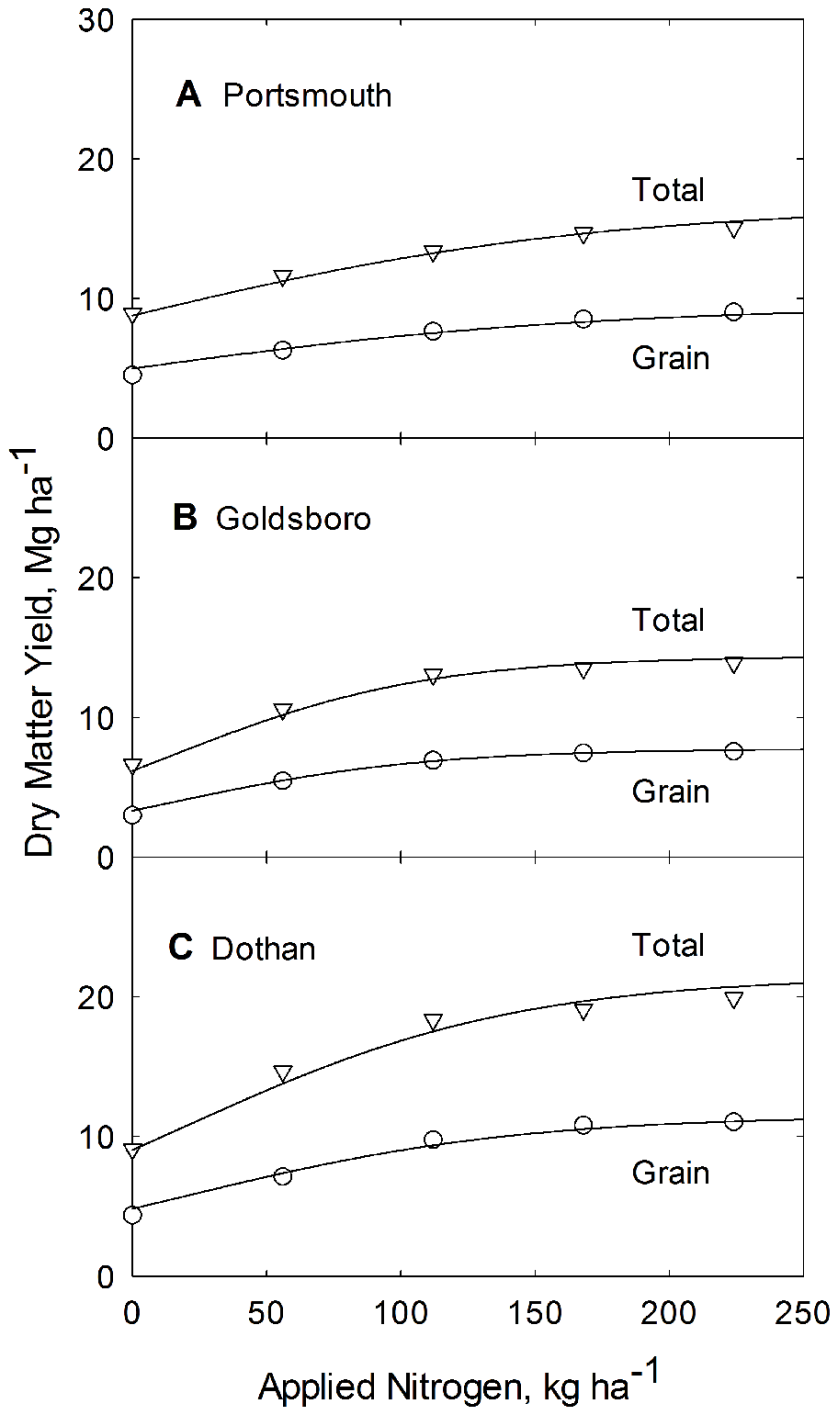
Dependence of grain and total plant biomass production on applied N for corn grown at the Plymouth (A), Kinston (B), and Clayton (C) experiment stations in North Carolina. Curves are constructed from Eq. 3 and from parameters listed in Table 2.

Grain and whole plant biomass production versus applied N is shown in Figure 2 for all three soil types. In general there is good agreement between the model line and the data. The resulting biomass model lines (depicted in Figure 2), are generated from Eq. (3), using parameter values for *b* and *c* from Table 2 and values for *A_g_* and *A_t_* found in both Table 3. Equation specific Non-linear Coefficient of Determination values and Error Sum of Squares are provided in Table 4.

**Figure 3:**
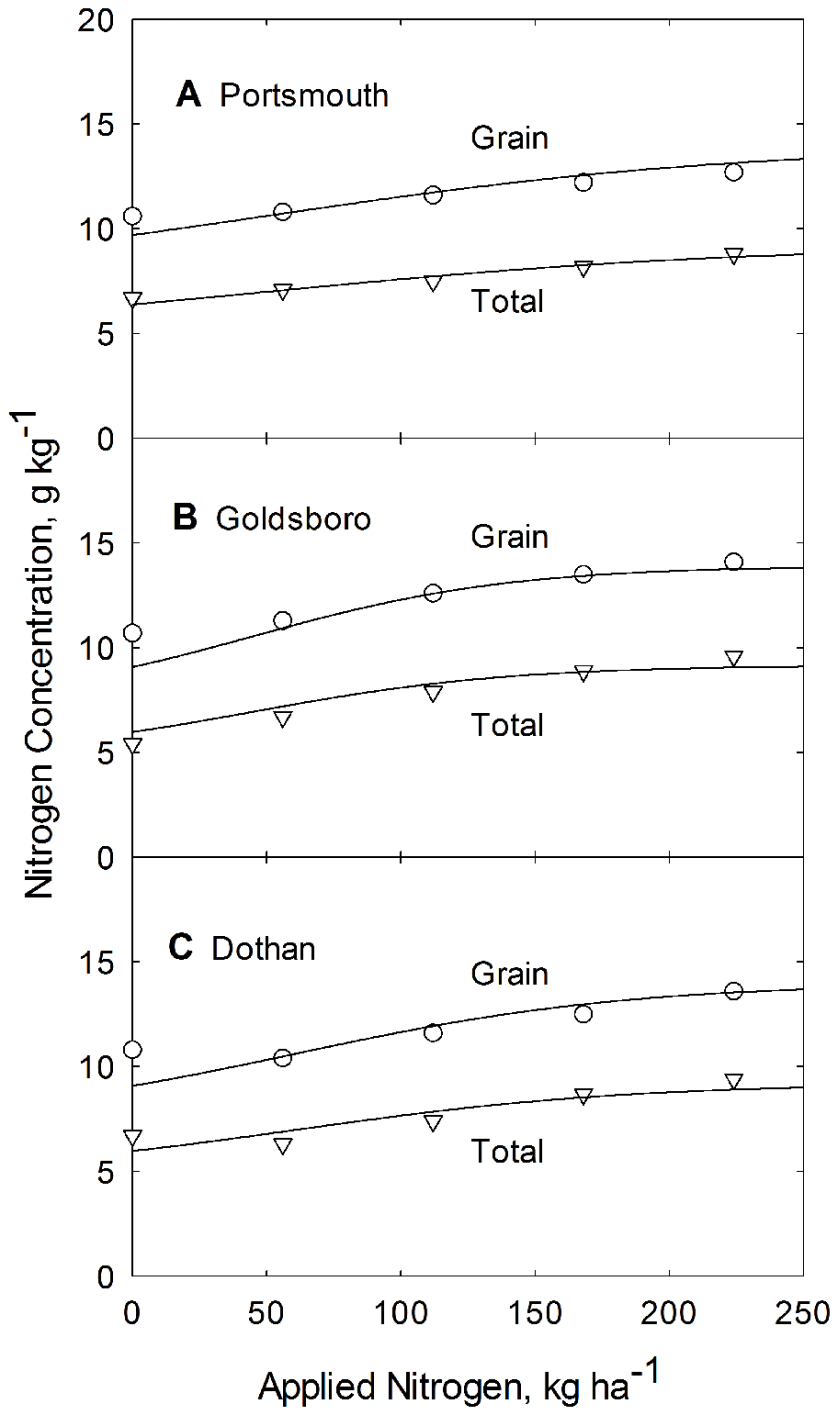
Dependence of grain and total plant N concentration on applied N for corn grown at the Plymouth (A), Kinston (B), and Clayton (C) experiment stations in North Carolina. Curves are constructed from Eq. 7 and from parameters listed in Table 2.

N concentration dependence on applied N is shown in Figure 3 for all three soil types. The resulting N concentration model lines (depicted in Figure 3), are generated from Eq. (7), using parameter values for *b*, *b_N_* and *c* from Table 2 and values for *N_cm g_* and *N_cm t_* from Table 6.

**Table 6:** Parametric factors invariant to site attributes, including soil type.

Plant Fraction	Parameter	Estimate
Both	*Δb*, kg ha^−1^	0.660
Grain	*N_cm g_*, g kg^−1^	14.0
Total Plant	*N_cm t_*, g kg^−1^	9.18
Grain	*N_cl g_*, g kg^−1^	7.23
Total Plant	*N_cl t_*, g kg^−1^	4.75
Grain	*N_cm t_*_−_ *N_cl t_ = A_Nt_/Y_mt_*, g kg^−1^	6.75
Total Plant	*N_cm g_*_−_ *N_cl g_ = A_Ng_/Y_mg_*, g kg^−1^	4.43

**Figure 4:**
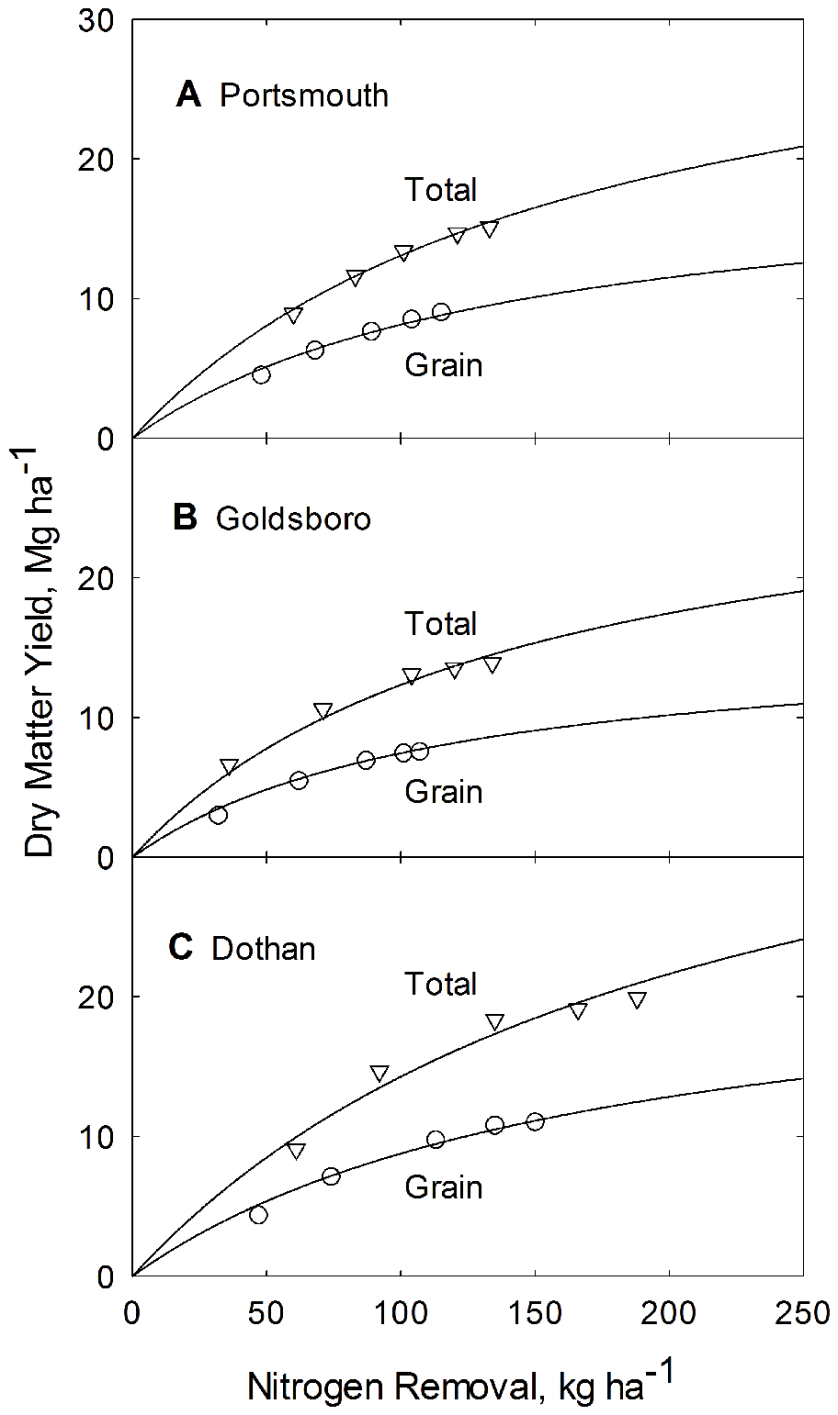
Dependence of grain and total plant biomass production on N uptake for corn grown at the Plymouth (A), Kinston (B), and Clayton (C) experiment stations in North Carolina. Curves are constructed from Eq. 2 and from parameters listed in Table 3.

The phase relationship between biomass production and N uptake for the corn grain and the whole plant is represented by Figure 4 for each of the three soils. The resulting biomass – N uptake phase model lines (depicted in Figure 4), are generated from Eq. (2), using parameter values for *k_Ng_*, *k_Nt_*, *Y_mg_* and *Y_mt_* found in Table 5.

**Table 5:** Standard phase model parameters for corn grain and total plant biomass production and for corn grain and total plant N uptake.

Soil	Parameter	Plant Fraction	Estimate
Dothan	*k_Ng_*, kg ha^−1^	Grain	157
	*k_Nt_*, kg ha^−1^	Total Plant	193
	*Y_mg_*, Mg ha^−1^	Grain	22.7
	*Y_mt_*, Mg ha^−1^	Total Plant	42.6
Goldsboro	*k_Ng_*, kg ha^−1^	Grain	104
	*k_Nt_*, kg ha^−1^	Total Plant	125
	*Y_mg_*, Mg ha^−1^	Grain	15.1
	*Y_mt_*, Mg ha^−1^	Total Plant	27.6
Portsmouth	*k_Ng_*, kg ha^−1^	Grain	122
	*k_Nt_*, kg ha^−1^	Total Plant	142
	*Y_mg_*, Mg ha^−1^	Grain	17.7
	*Y_mt_*, Mg ha^−1^	Total Plant	31.4

**Figure 5:**
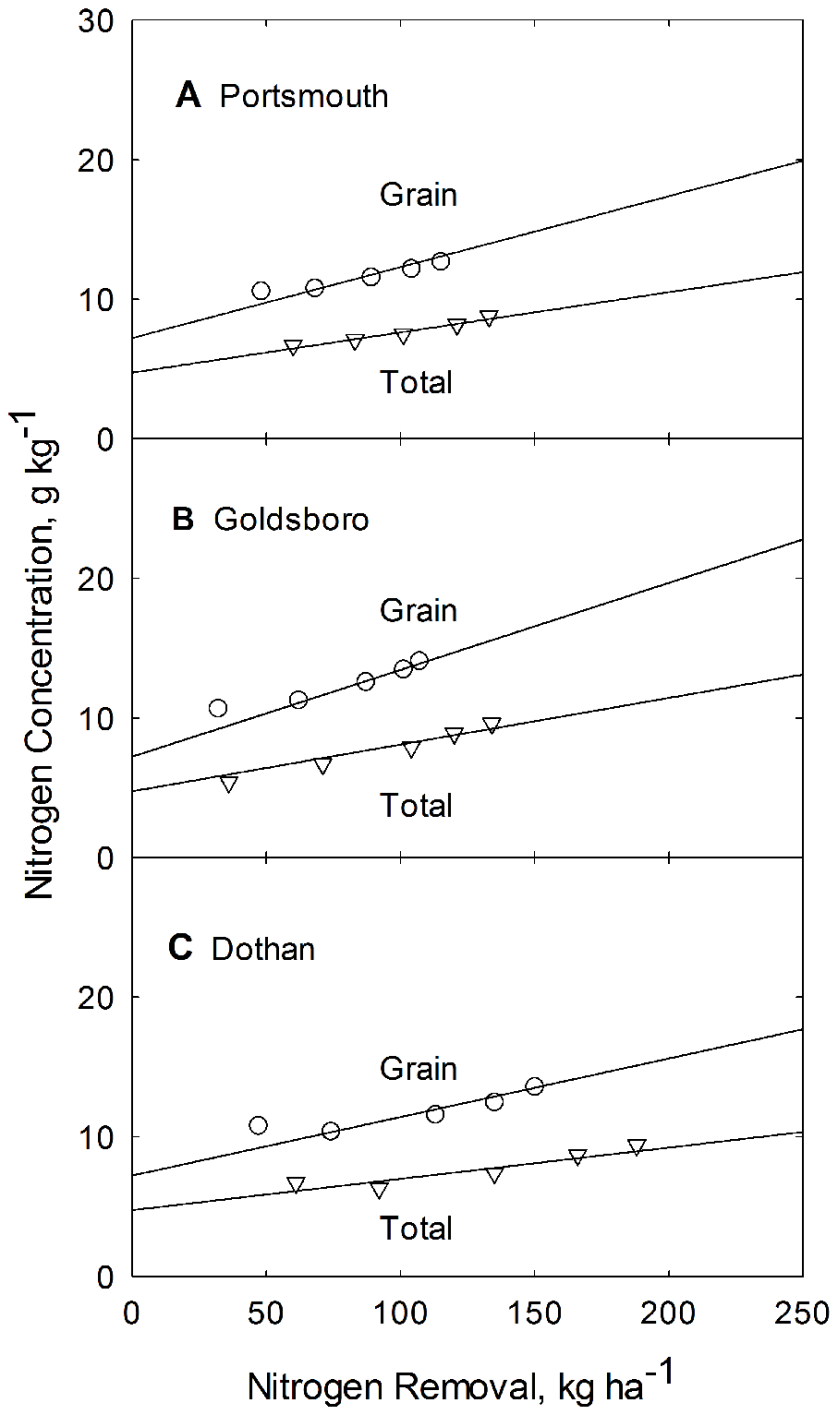
Dependence of grain and total plant N concentration on N uptake for corn grown at the Plymouth (A), Kinston (B), and Clayton (C) experiment stations in North Carolina. Curves are constructed from Eq. 11 and from parameters listed in Table 3.

The phase relationship between N Concentration and N uptake for the grain and the whole plant is represented by Figure 4 for each of the three soils. The resulting between N Concentration – N uptake phase model lines (depicted in Figure 5), are generated from Eq. (11), using parameter values for *k_Ng_*, *k_Nt_*, *Y_mg_* and *Y_mt_* found in Table 5.

## Discussion

From this analysis it is concluded that there are aspects of the ELM that are invariant with respect to both soil type and water availability for a given variety of annual crop propagated by seeding and harvested at the same relative age. This analysis has shown, for the Kamprath N-rate study conducted on corn in North Carolina [22] that both the difference between N uptake intercept parameter and the biomass intercept parameter, *Δb*, and the maximum N concentration, *N_cm_*, are in fact invariant with respect to the crop’s surrounding environmental conditions. From the model, these facts lead to the conclusion that both the upper limit N concentration, *N_cm_*, and the lower limit N concentration, *N_cl_*, are both invariant with respect to soil type and water availability in the study analyzed. This further suggests that the *N_cm_* and *N_cl_* parameters are of more importance to the model. While other parameters, such as
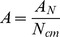
(24)




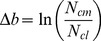
(25)




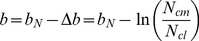
(26)




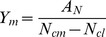
(27)and
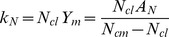
(28)can be rewritten, to show the significance that upper and lower limit concentrations have in each parameter and ultimately seasonal plant response to nutrient application. Having upper and lower limits to plant nutrient concentration corresponds with plant physiology. Without a minimum level of a given required nutrient, there can be no yield, seasonal or otherwise. There should also be a maximum concentration that can be approached, as there should be diminishing yield increases as higher agronomic rates are applied, or there would be unbounded growth.

If this invariance with respect to soil type and water availability holds for all crops propagated by seeding, the model could be written in terms of parameters that have measurable physiological significance and could give further insight into relationships that govern plant development and nutrient removal. Initial evidence appears promising that perennial crops, such as ryegrass (*Lolium perenne* L.), when held to comparably the same seasonal management practices also exhibit very nearly the same conclusions with regard to both constant values of the *N_cm_* and *N_cl_* parameters [25].

Given that N uptake and biomass production can be described by five parameters, and if two are invariant to all but crop type and season length, the model reduces to three parameters (*A_N_*, *b_N_*, and *c*) when a crop and season length are chosen. From Overman & Scholtz [26] the logistic response originates within the soil’s buffering capacity for P and for K, and the *c* parameter remains the same from the plant extractable logistic response to nutrient uptake logistic response, and to biomass production. It is here assumed that the *c* parameter for applied N also originates as the rate response parameter for the soil’s buffering capacity of N. The *c* parameter can be modified by plant population [11]. A Future step should be to analyze various field studies to catalogue soil physical and chemical characteristics and the resulting impact on the *c* parameter. Mathematically *b_N_* represents shifting parameter which in conjunction with the *c* parameter as
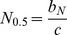
(29)


*N*_0.5_ represents the effective level of N necessary to achieve peak N uptake efficiency [10], [16], [17]. Ultimately, for environmental considerations, setting applied rate of N to the peak uptake level will result in the most N removed per unit N applied. Plus, provided the difference between *b* and *b_N_* is greater than 0, then the yield will be on the upper portion of the logistic biomass curve to yield
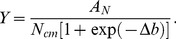
(30)


The *b_N_* parameter is affected by changes in plant population [11] and is also influenced by crop type [8], [25]. The remaining parameter, *A_N_*, is a linear parameter that is affected by the various environmental conditions, the crop type, the soil type, and various management practices [5]–[20]. Given Eq. (30), knowing the invariant Δ*b* value for a given crop, and having a reasonable estimate for the background level of N already present in the soil, represented by Eq. (29), exists the beginning of a framework for a more reasonable and more sustainable nutrient management guide. Further analyses are being conducted to verify these findings with other annual propagated by seeding and with perennial crops.
